# The Complete Genome Sequence of the *Staphylococcus* Bacteriophage Metroid

**DOI:** 10.1534/g3.120.401365

**Published:** 2020-07-29

**Authors:** Adele Crane, Joy Abaidoo, Gabriella Beltran, Danielle Fry, Colleen Furey, Noe Green, Ravneet Johal, Bruno La Rosa, Catalina Lopez Jimenez, Linh Luong, Garett Maag, Jade Porche, Lauren Reyes, Aspen Robinson, Samantha Sabbara, Lucia Soto Herrera, Angelica Urquidez Negrete, Pauline Wilson, Kerry Geiler-Samerotte, Susanne P. Pfeifer

**Affiliations:** * School of Life Sciences, Arizona State University, Tempe, AZ; † Center for Evolution and Medicine, Arizona State University, Tempe, AZ; ‡ Center for Mechanisms of Evolution, Arizona State University, Tempe, AZ

**Keywords:** bacteriophage, *Myoviridae*, *de novo* assembly, gene annotation

## Abstract

Phages infecting bacteria of the genus *Staphylococcus* play an important role in their host’s ecology and evolution. On one hand, horizontal gene transfer from phage can encourage the rapid adaptation of pathogenic *Staphylococcus* enabling them to escape host immunity or access novel environments. On the other hand, lytic phages are promising agents for the treatment of bacterial infections, especially those resistant to antibiotics. As part of an ongoing effort to gain novel insights into bacteriophage diversity, we characterized the complete genome of the *Staphylococcus* bacteriophage Metroid, a cluster C phage with a genome size of 151kb, encompassing 254 predicted protein-coding genes as well as 4 tRNAs. A comparative genomic analysis highlights strong similarities – including a conservation of the lysis cassette – with other *Staphylococcus* cluster C bacteriophages, several of which were previously characterized for therapeutic applications.

Pathogens of the genus *Staphylococcus*, known for their ability to evade the human immune system, are an important public health concern causing a multitude of community-acquired infections ranging from food poisoning to skin lesions and life-threatening sepsis (Pollitt *et al.* 2018). As *Staphylococcus* largely reproduces clonally, much of the genetic diversity among strains stems from horizontal gene transfer through bacteriophages. Thereby, the acquisition of novel genes may not only aid adaptation of a bacterial strain to novel environments (Xia and Wolz 2014), but it can also increase pathogenicity. Bacteriophages play an important role in bacterial pathogenesis (Deghorain *et al.* 2012) as they encode for many known staphylococcal virulence factors (see review by Malachowa and DeLeo 2010). Moreover, bacteriophages can mediate the mobilization and transfer of genomic pathogenicity islands (Xia and Wolz 2014). On the other hand, virulent bacteriophages, which lyse their host cell after successful reproduction, also represent promising new avenues for the treatment of antibiotic-resistant *Staphylococcus* infections through phage therapy (Moller *et al.* 2019).

Approximately 10^30^ bacteriophages are estimated to exist on our planet (Rohwer 2003), however much of their diversity remains under-sampled and therefore uncharacterized. Several *Staphylococcus* phages (order: *Caudovirales*; *i.e.*, tailed dsDNA phages) have been isolated and sequenced (*e.g.*, Kwan *et al.* 2005; Deghorain *et al.* 2012; Oliveira *et al.* 2019). Historically, *Staphylococcus* phages were grouped according to their lytic activity and serology; specifically, their reaction to (among others) polyclonal antiserum (Rountree 1949; Rippon 1952, 1956). In contrast, modern phage classification systems are based on either: 1) morphology (determined using transmission electron microscopy), categorizing *Myoviridae* (long, contractile tail; group A), *Siphoviridae* (long, non-contractile tail; group B), and *Podoviridae* (short tail; group C) (Ackermann 1975; Brandis and Lenz 1984); 2) genome size, categorizing class I (<20kb), class II (∼40kp), and class III (>125kb) (Kwan *et al.* 2005); or 3) gene homology (Goerke *et al.* 2009; Kahánková *et al.* 2010; McCarthy *et al.* 2012), with phages of like category generally being more closely related to one another (Kwan *et al.* 2005). In one of the largest *Staphylococcus* phage genomic studies published to date, Oliveira *et al.* (2019) used a comparative evolutionary approach to group *Staphylococcus* phages according to their gene content: cluster A (morphologically *Podoviridae*; genome size: 16-18kb), cluster B (a diverse cluster consisting of mostly temperate phages; genome size: 39-48kb), cluster C (morphologically *Myoviridae*; genome size: 127-152kb), and cluster D (morphologically *Siphoviridae*; genome size: 89-93kb). Based on predicted sequence similarities of protein families (phams), the authors further subdivided *Staphylococcus* phages into 27 subclusters (A1-A2, B1-B17, C1-C6, and D1-D2), members of which exhibit similar morphology and genomic features (*i.e.*, genome size, GC-content, and number of genes; Oliveira *et al.* 2019). In contrast to the usually temperate *Siphoviridae*, most *Myoviridae* and *Podoviridae* experimentally characterized to date exhibit a lytic life cycle. Lytic phages destroy their host cells, making them interesting candidates for phage therapy (Xia and Wolz 2014).

Here, we report the complete genome sequence of the *Staphylococcus* bacteriophage Metroid, a *Myoviridae* sequenced as part of HHMI’s SEA-PHAGES program – an ongoing effort to systematically characterize bacteriophages and their relationship to their (often pathogenic) bacterial hosts. A comparative genomic analysis highlights strong similarities with other *Staphylococcus* cluster C bacteriophages, several of which were previously characterized for therapeutic applications (Vandersteegen *et al.* 2011; Gill 2014; Leskinen *et al.* 2017; Ajuebor *et al.* 2018; Philipson *et al.* 2018).

## Materials And Methods

Sample collection, isolation, purification, amplification, and phage characterization followed the HHMI SEA-PHAGES Phage Discovery Guide (https://seaphagesphagediscoveryguide.helpdocsonline.com/home; last accessed 2020/04/30), with modifications indicated below. Media and reagent preparation followed the HHMI SEA-PHAGES recipe cards in the Phage Discovery Instructors Guide (https://phagediscoveryinstructorguide.helpdocsonline.com/appendix-b-recipe-cards; last accessed 2019/11/30). Library preparation, sequencing, assembly, and gene annotation followed the HHMI SEA-PHAGES Phage Genomics Guide (https://seaphagesbioinformatics.helpdocsonline.com/home; last accessed 2020/04/30).

### Sample collection and isolation

To locate phage, ∼50 soil samples were collected from various locations in Arizona and plaque assays were performed on the sample filtrates. Most samples did not produce phage that could infect the host bacteria. The sample that produced Metroid was collected from a shaded and well-irrigated garden on Arizona State University’s Tempe campus (33.417708N, 111.935974W; ambient temperature 37.7°). The soil was loosely packed into half of a 15 mL conical tube and stored at 4° until phage isolation and a plaque assay were performed. In order to isolate bacteriophages, the sample was submerged in 10 mL PYCa liquid media (1 g/L of yeast extract, 15 g/L of tryptone, 4.5 mM CaCl_2_, 0.1% dextrose, 10 μg/mL cycloheximide), vortexed for one minute, and placed in a shaking incubator at room temperature for 30 min. This sample was then centrifuged at 4500 rpm for four minutes and filter-sterilized with a 0.22 μm syringe filter. A 250 μL sample of this filtrate was mixed with 250 μL of host bacteria. The host bacteria was isolated as a contaminant from frozen cultures of *Arthrobacter globiformis*. We suspect it to be of the genus *Staphylococcus* given that it possesses phage known to reside in this genus. Before mixing with the filtered soil sample, the host bacteria had been grown to saturation in PYCa and stored at 4°. After a ten minute incubation at room temperature, the 500 μL of phage plus bacteria was added to 4.5 mL molten PYCa top agar (60°) and immediately plated on a PYCa agar plate which was incubated for 48 hr at 37°.

### Purification and amplification

Clear plaques appeared on the PYCa plates after 48 hr and were ∼3 mm in diameter. One plaque was picked with a sterile pipette tip, and phage were resuspended in phage buffer (10 mM Tris, 10 mM MgSO_4_, 68 mM NaCl, ddH2O, 1 mM CaCl_2_), and a series of six 10-fold serial dilutions were performed. Each dilution was inoculated with 250 µL of host bacteria and incubated at room temperature for ten minutes. Each dilution was plated with 4.5 mL PYCa top agar and incubated at 37° for 48 hr. A plaque from the plate representing the 10^−2^ dilution was selected to complete two additional rounds of purification through subsequent dilutions and plaque assays. For each purification, we chose to pick plaques from a ‘countable’ plate, on which plaques were separated enough to suggest that each grew from a single phage particle (typically a countable plate had 30 to 300 plaques).

Once purified, we amplified the phage to obtain a titer greater than 1x10^9^ PFU/mL which would provide enough DNA for genome sequencing. A plate containing numerous purified phage plaques was flooded with 8 mL of phage buffer and set at room temperature for an hour to yield a phage lysate. The lysate was collected in a 15 mL tube and centrifuged at 8000 rpm for four minutes then filtered through a 3 mL syringe with a 0.22 µL filter. 10-fold serial dilutions were made with the collected lysate for amplification. A spot titer was made with the undiluted lysate as well as 10^−1^ to 10^−10^ lysate dilutions. Based on counting the number of plaques formed by each lysate in the spot titer assay, the 10^−8^ dilution was selected as the best candidate to produce a countable plate. A full titer plate was prepared with the 10^−7^, 10^-8,^, and 10^−9^ dilutions. The titer calculated from the full titer assay was 2.65x10^10^ PFU/mL.

### Phage characterization – DNA extraction

DNA extraction was performed on the phage lysate using the Wizard DNA Clean-Up kit (Promega) with minor modifications. 5 µL of nuclease mix (150 mM NaCl, ddH_2_O, 0.25 mg/mL DNase 1, 0.25 mg/mL RNase A, 50% glycerol) was added to 1 µL of lysate and mixed by inversion. The solution was incubated at 37° for ten minutes. 15 µL of 0.5 M EDTA and 1 µL of 20 mg/mL Proteinase K were added to the solution and incubated at 37° for 20 min. 2 mL of Wizard DNA Clean-Up resin (Promega) was added to the solution and mixed by inversion for two minutes. The solution was syringed-filtered through two Wizard Genomic DNA columns (Promega) and then washed three times with 80% isopropanol. The columns were twice spun in a centrifuge at top speed for two minutes and then placed in a 90° heat block for one minute. 50 µL of ddH_2_O was used for elution. Final elutes were combined for 100 µL of total DNA extract. A Nanodrop ND 1000 was used to determine a DNA concentration of 114.9 ng/µL.

### Phage characterization – Transmission Electron Microscopy

A high-titer lysate was made up for Transmission Electron Microscopy (TEM) by spinning 100 µL of phage lysate in a 4° Centrifuge at top speed for 22 min. The supernatant was removed and the pellet was resuspended in 10 µL of phage buffer. The high-titer lysate then underwent TEM preparation by negatively staining the virus particles. Specifically, isolated particles were adhered to a 300-mesh carbon-formvar grid for one minute, followed by staining with 1% aqueous uranyl acetate for 30 sec. Images were acquired using a Philips CM12 TEM operated at 80kV and equipped with a Gatan model 791 CCD camera.

### Library preparation, sequencing, and de novo assembly

A sequencing library was prepared from genomic DNA by using a NEB Ultra II FS kit with dual-indexed barcoding and sequenced on an Illumina MiSeq, yielding a total of 901,246 single-end 150bp reads (>895X coverage). Quality control checks using FastQC v.0.11.7 (http://www.bioinformatics.babraham.ac.uk/projects/fastqc; last accessed 2020/04/30) indicated that the data were of high quality (*i.e.*, no adapters were present and base quality scores were >30, equivalent to an error rate of <0.1%; Figure S1). Consequently, no adapter contaminations were trimmed off of the 3′-end of the reads by scythe v.0.991 (a Naive Bayesian approach to detect and remove contamination) and sickle v.1.33 (a tool for quality-based read trimming) flagged only ∼0.1% of the reads for containing base qualities <20. With no significant changes to our dataset, additional read processing prior to assembly was thus deemed unnecessary. Following Russell (2018), reads were *de novo* assembled using Newbler v2.9, resulting in a single linear contig of size 150,935bp, which was checked for completeness, accuracy, and phage genomic termini using Consed v.29 (Gordon *et al.* 1998). All software was executed using default settings.

### Genome annotation

Annotation was performed using DNA Master v.5.23.3 (http://cobamide2.bio.pitt.edu; last accessed 2020/04/30). Putative protein-encoding open reading frames (genes) were identified using Glimmer v.3.0 (Delcher *et al.* 1999) and GeneMark v.2.5 (Lukashin and Borodovsky 1998) with AUG (methionine), UUG and CUG (leucine), GUG (valine), and AUA (isoleucine) as start codons. Using annotated bacteriophage sequences from public databases, functional assignments were made with Blastp v.2.9 (Altschul *et al.* 1990) – both within the DNA Master environment and within NCBI to take advantage of the Conserved Domain Database (Marchler-Bauer *et al.* 2015) – as well as with HHPred (Söding *et al.* 2005) which, in addition to sequence similarity, also compares putative three-dimensional protein structures. TMHMM2 (Krogh *et al.* 2001) and SOSUI (Hirokawa *et al.* 1998) were used to identify membrane proteins. tRNAs were annotated using Aragon v.1.1 (included in DNA Master) and v.1.2.38 (Laslett and Canback 2004) as well as tRNAscan-SE v.2.0 (Lowe and Eddy 1997). All software was executed using default settings.

### Comparative genomics analysis

Due to their similar length, number of genes and tRNAs, as well as GC-content, the genomes of the phages IME-SA1, IME-SA2, ISP (Vandersteegen *et al.* 2011), JA1 (Ajuebor *et al.* 2018), K (Gill 2014), vB_SauM_0414_108 (Philipson *et al.* 2018), and vB_SauM-fRuSau02 (Leskinen *et al.* 2017) were downloaded from GenBank (Table 1) to create a database of *Staphylococcus* cluster C1 phages (Oliveira *et al.* 2019) using PhamDB (Lamine *et al.* 2016). This custom database was used for all subsequent comparative analyses. First, a multiple sequence alignment was performed utilizing Kalign v.1.04 (Lassmann and Sonnhammer 2005) to produce a neighbor-joining tree. Second, dotplots, comparing the relatedness of different nucleotide sequences, were generated in 10bp sliding windows using Gepard v.1.40 (Krumsiek *et al.* 2007). Lastly, the database was loaded into Phamerator (Cresawn *et al.* 2011) to visually compare phage genomes.

**Table 1:** FEATURES OF METROID AND THE SEVEN *STAPHYLOCOCCUS* CLUSTER C1 PHAGES USED FOR COMPARATIVE ANALYSES

name	length	# genes	# tRNAs	GC-content	host	GenBank accession number	reference
Metroid	150,935	254	4	30.40	*S*. spp.*^a^*	MT411892.1	this study
IME-SA1	140,218	209	4	30.33	*S. aureus*	KP687431.1	unpublished
IME-SA2	140,906	212	4	30.33	*S. aureus*	KP687432.1	unpublished
ISP	138,339	215	4	30.42	*S. aureus*	FR852584.1	Vandersteegen *et al.* 2011
JA1	147,135	233	4	30.25	*S. aureus*	MF405094.1	Ajuebor *et al.* 2018
K	148,317	233	4	30.39	*S. aureus*	NC_005880.2	Gill 2014
vB_SauM_0414_108	151,627	249	4	30.39	*S. aureus*	MH107769.1	Philipson *et al.* 2018
vB_SauM-fRuSau02	148,464	236	4	30.22	*S. aureus*	MF398190.1	Leskinen *et al.* 2017

a presumptive.

### Data availability

Figure S1 depicts the quality control checks of the raw read data using FastQC. Whole genome sequencing data are available through NCBI’s Sequence Read Archive (BioProject accession number PRJNA640949) and the annotated genome assembly is available through GenBank (accession number MT411892.1). Supplemental material available at figshare: https://doi.org/10.25387/g3.12585056.

## Results And Discussion

**Figure 1:**
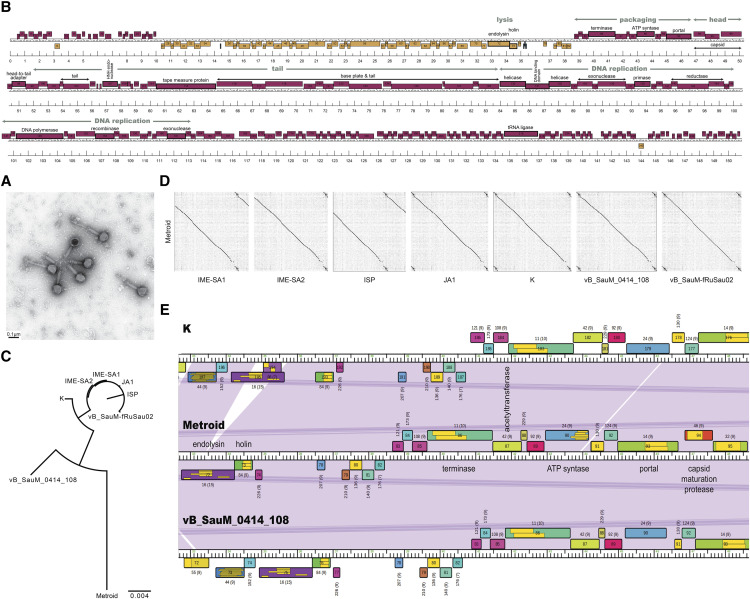
CHARACTERIZATION OF METROID AND ITS RELATEDNESS TO OTHER *STAPHYLOCOCCUS* CLUSTER C PHAGES. a) Transmission electron microscopy image showing Metroid’s morphology. b) Metroid’s genome contains 254 predicted protein-coding genes as well as 4 tRNAs; total genome size: 151kb including the ∼10kb terminal repeat. The majority of genes are transcribed on the forward strand as shown in pink; genes transcribed on the reverse strand are highlighted in orange; tRNAs in blue. Functionally related genes are organized into distinct modules (highlighted in gray). c) Neighbor-joining tree and d) dotplot of Metroid and seven previously described *Staphylococcus* bacteriophages (Table 1). e) Genes in the lysis cassettes as well as in the packaging module show a strong conservation between Metroid and two closely-related *Staphylococcus* phages, K (Gill 2014) and vB_SauM_0414_108 (Philipson *et al.* 2018). Genes are labeled with their putative function, with genes belonging to the same protein family (pham) depicted in the same color. Purple coloring between genomes highlights regions of high nucleotide similarity (*i.e.*, a BLAST e-value of 0).

The complete genome sequence of the *Staphylococcus* bacteriophage Metroid was sequenced and annotated (see “Materials and Methods” for details). The *Myoviridae* morphology (*i.e.*, an icosahedral capsid [diameter: 100nm] enclosing the double-stranded DNA attached to a long, contractile tail [length: 108nm]; Figure 1a) as well as the genome size of 151kb (including the ∼10kb terminal repeat) suggests that Metroid belongs to the *Staphylococcus* phage cluster C. Metroid’s genome has a GC-content of 30.40%, similar to those of previously published *Staphylococcus* phages (27.98–34.96%) (Kwan *et al.* 2005; Deghorain *et al.* 2012; Oliveira *et al.* 2019). The tightly-packed genome contains 254 predicted protein-coding genes as well as 4 tRNAs, most of which are transcribed on the forward strand (Figure 1b). This corresponds to a gene density of 1.68 genes/kb – on the upper end of the range previously reported for cluster C phages (164-249 genes; 0-5 tRNAs; 1.25-1.64 genes/kb) (Oliveira *et al.* 2019). Although the overall gene coding potential of Metroid is 89.42%, only 26 of the 254 predicted proteins could be assigned a putative function. The majority of predicted proteins are either conserved but of no known function (170 out of 254), membrane proteins (22), or unique (*i.e.*, without a match to any of the queried databases; 1). As previously observed in other *Staphylococcus* phages (Kwan *et al.* 2005), functionally related genes are organized into distinct modules (*e.g.*, distinct head and tail modules connected by a head-to-tail adapter; Figure 1b), the respective order of which is largely conserved across phages of the same category.

Complementing the classification by morphology and genome size, comparative genomic analysis with seven *Staphylococcus* subcluster C1 phages highlights a strong relatedness on the sequence level (Figures 1c,d) and thus, provides additional evidence for the assignment of Metroid to cluster C. Metroid is most closely related to vB_SauM_0414_108 (Figures 1c,d) – a phage discovered as part of a recent effort proposing a guideline and standardized workflow to submit phages to the Federal Drug Administration to be considered as potential future treatments of bacterial infections (Philipson *et al.* 2018). More generally, genes in the lysis cassettes show a strong conservation between Metroid and the closely-related *Staphylococcus* cluster C phages, including phages K (Gill 2014) and vB_SauM_0414_108 (Philipson *et al.* 2018), which both share 99% amino acid identity with Metroid for endolysin and >97% amino acid identity for holin (Figure 3e). Both phages were previously characterized for therapeutic research, suggesting that Metroid might be a suitable candidate for future phage therapies.
